# How Advanced Artificial Intelligence Technologies Shape Drug–Drug and Drug–Target Interaction Modeling

**DOI:** 10.1002/advs.75819

**Published:** 2026-05-29

**Authors:** Xin Sun, Tong Wang

**Affiliations:** ^1^ State Key Laboratory of Membrane Biology, Beijing Frontier Research Center for Biological Structure, Tsinghua‐Peking Center for Life Sciences, Center for Life Sciences and Artificial Intelligence, School of Life Sciences Tsinghua University Beijing China

**Keywords:** drug–drug interaction, drug–target interaction, deep learning, drug discovery

## Abstract

Drug molecular interactions, including drug–drug interactions (DDIs) and drug–target interactions (DTIs), are critical for drug discovery and clinical safety, increasingly propelled by artificial intelligence (AI) technologies. Although previously treated as separate domains, DDIs and DTIs are highly interconnected in terms of biological mechanisms and model design. To foster their co‐evolution, this review provides a comprehensive landscape of drug molecular interaction modeling by first summarizing the advanced AI technologies across various prediction tasks in both domains. Then, the parallel development paths are examined in core architecture, feature engineering, and model learning paradigms, highlighting the convergence in patterns of feature engineering and trends of model design. Furthermore, the key challenges, such as insufficient generalizability and shortcut learning, are identified and evaluated through quantitative experiments, and future directions are proposed for building unified models to leverage AI in accelerating drug discovery and therapeutics design.

## Graphical abstract

This review explores the convergence of artificial intelligence technologies in modeling drug–drug and drug–target interactions. By evaluating advanced feature engineering, architectural innovations, and learning paradigms reveals shared evolutionary trends and critical challenges, such as cold‐start settings and shortcut learning. Bridging these distinct domains, the article proposes unified, physics‐informed frameworks to accelerate reliable drug discovery and rational therapeutics design.

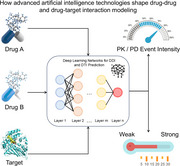

## 1 Introduction

The traditional drug discovery and development pipelines are time‐consuming and resource‐intensive, marked by high attrition rates [1, 2]. A key challenge lies in effectively capturing the pharmacological and physicochemical profiles of existing compounds, including drug molecules and protein targets, from their interactions leading to expected molecular mechanisms to those resulting in unexpected side effects. In this scenario, two predictive tasks take the major attention: (1) the prediction of drug–drug interactions (DDIs); and (2) the prediction of drug–target interactions (DTIs). DDIs describe the phenomenon where the efficacy, metabolism, or toxicity of one drug is altered by the co‐administration of another, directly impacting the safety and effectiveness of combination therapies [3]. DTIs are crucial for understanding the action mechanisms of various drugs, identifying new drug–target pairs, and elucidating the off–target effects [4]. As the artificial intelligence technologies and deep learning are rapidly boosting in the scientific research field, the deep neural networks have become powerful alternatives for modeling molecular interactions for drug discovery [5, 6, 7, 8]. These computational methods have been noticeably advancing DDI and DTI predictions from early‐stage approaches based on similarity assumptions and handcrafted feature engineering [9, 10, 11, 12] to recently elaborately designed model architectures like Graph Neural Networks (GNNs) [13, 14, 15], Transformers [16, 17], and Large Language Models (LLMs) [18, 19]. These models learn complex interaction patterns from the molecular sequences, structures and broader biomedical knowledge networks (BKGs) of drugs and target proteins [20, 21].

Despite remarkable advancements in DDI and DTI predictions by deep learning models, the shared patterns and trends of these two tasks have been largely overlooked and underutilized. Most models are designed for either DDI prediction or DTI prediction, respectively. However, the two tasks are highly related and thus can be described in a unified way. The common characteristics can be explained from two aspects: the shared biological mechanisms in metabolism and signal pathway, and the similar trends in model architecture design. The former is reasonable at both the pharmacokinetic and pharmacodynamic levels. For example, in pharmacokinetic level, when one drug acts as a perpetrator, it could inhibit or induce a metabolic enzyme, such as those in the Cytochrome P450 family [22, 23]. This modulation of enzyme function is a DTI effect, which then directly alters the metabolic clearance of a co‐administered drug as a victim in turn, leading to a typical plasma concentrations that manifest as a DDI effect, such as toxicity or therapeutic failure [24, 25]. Similarly, a DDI effect on pharmacodynamic level could occur as direct competition, where two drugs vie for the same binding site on a shared molecular target or bind to different targets that are components of the same signal pathway [26, 27]. Then, resulting from these biological mechanism similarities, DDI prediction and DTI prediction exhibit a similiar developing trend on model design. For the input features, both fields have moved beyond single inputs to integrate heterogeneous BKGs. Furthermore, the model architectures have evolved from simple neural networks to GNNs, Transformers, and further large‐scale foundation models [28, 29, 30, 31], aiming to capture complex structural and long‐range dependencies of molecular interactions. In addition, to address the persistent challenge of data scarcity, both domains are increasingly turning to self‐supervised [32, 33] and meta‐learning [34, 35] strategies during the model training stage.

It is worth noticing that AI‐driven DDI and DTI predictions also face the common challenges. First, insufficient generalizability remains a primary bottleneck [36, 37] in both fields. In the context of molecular interaction, generalizability refers to the capacity to maintain robust and accurate prediction performance under a cold‐start scenario, where the unseen and novel biological entities in the test set are strictly excluded from the training phase. Different from the high prediction accuracy under a warm‐start scenario, where the biological entities in the test set have similarity with those in the training phase, empirical evidence shows that both DDI and DTI models suffer from noticeable performance drops in cold‐start settings. This gap indicates that current models may insufficiently capture the intrinsic molecular interactions. Second, the shortcut learning phenomenon also exists in both domains. Instead of learning the biochemical compatibility between pairs of drugs or drug–target, the deep learning models tend to memorize high‐frequency drugs or targets that occur during model training. Consequently, they assign higher prediction scores to the entities with higher positive label rates in the training set, neglecting the underlying biological properties of the interacting pairs. Third, data quality and curation standards are critical for ensuring fair comparisons. However, existing studies employ divergent pipelines for data preprocessing and splitting. Lacking standardization results in inconsistent dataset quality and size, further leading to biased benchmark evaluations. Meanwhile, the inaccessibility of valuable private data also limits the opportunity to explore wider biochemical space. In addition, the other two factors are also crucial, i.e., the need to carefully handle model complexity with computational cost, and the necessity of establishing more comprehensive principles to enhance model interpretability.

Therefore, in this review, we investigate the co‐evolution of deep learning approaches for both DDI and DTI predictions. We demonstrate that, despite their different biological contexts, these two fields share fundamental algorithmic principles and confront the same critical bottlenecks. The following parts of this review are structured as follows: (1) First, we classified the tasks for drug molecular interaction predictions, i.e., outlining the specific scientific problem settings and input data standards for both DDI and DTI predictions; (2) Second, we systematically summarized the latest state‐of‐the‐art AI models for both domains, elucidating their input feature developments, comparing the model architectural innovations, and tracing the evolutions in model learning paradigms; (3) Third, we highlighted the shared patterns of feature engineering and trends of model design for DDI and DTI predictions; (4) Furthermore, we investigated the critical intersecting challenges, such as model generalizability for cold‐start scenarios and shortcut learning phenomenon. Notably, through quantitative analyses, we demonstrated these challenges are prevalent across different tasks and models; (5) Finally, we concluded by prospecting the future vision and providing potential solutions for future developments. Through this review, we aim to provide a clear vision and insight into the forefront of AI‐driven drug molecular interaction modeling, advancing, and accelerating drug discovery and therapeutics design.

## 2 Classification of Drug Molecular Interaction Predictions

**FIGURE 1:**
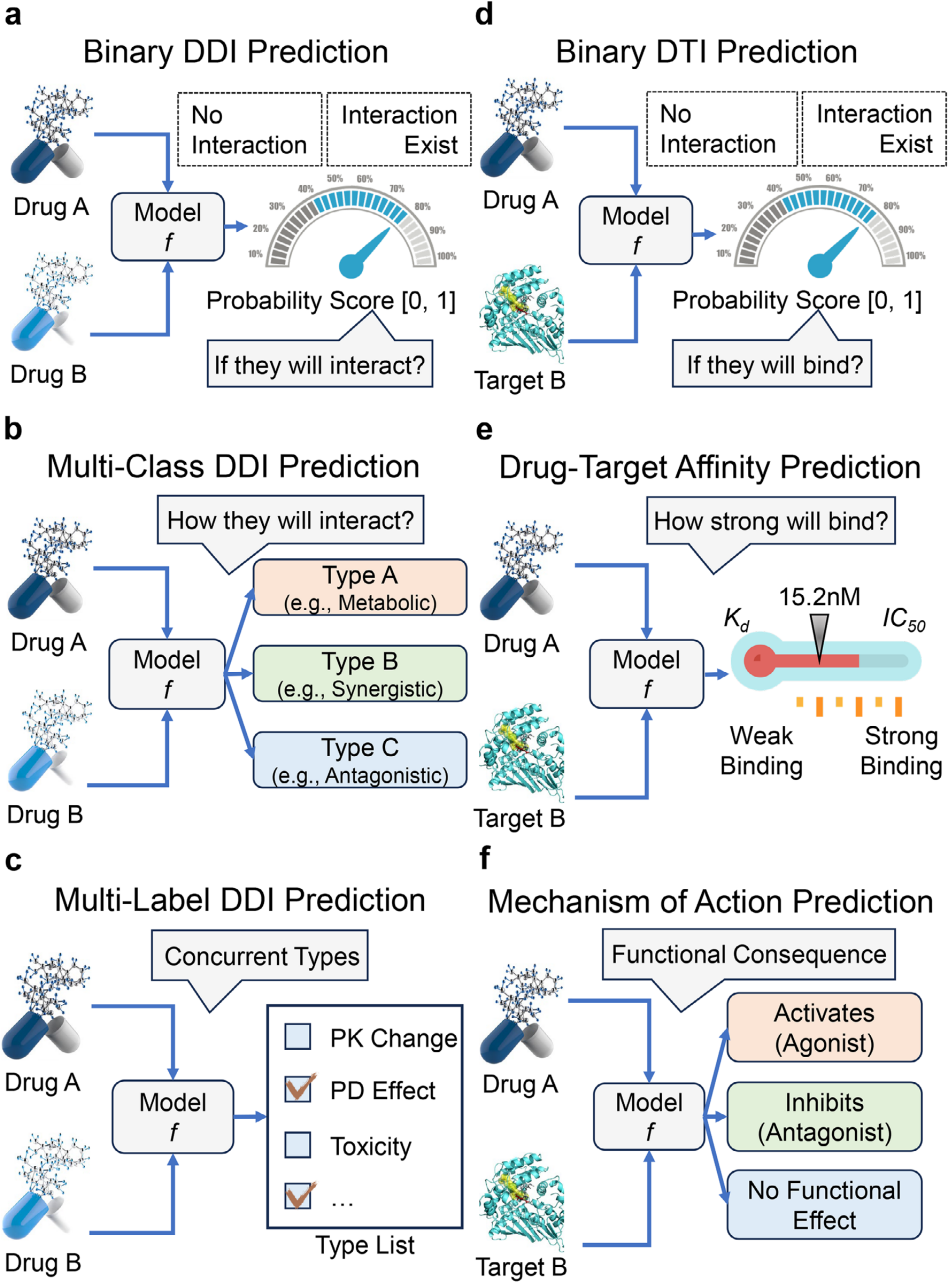
The schematic illustration of the prediction tasks for DDI and DTI predictions. The framework distinguishes between two major categories. (a–c), Illustrate the tasks in DDI prediction. The model f takes a pair of drug entities $\left\langle e_{i}^{\text{drug}},e_{j}^{\text{drug}}\right\rangle$ as input. (a) Binary DDI prediction determines the existence of an interaction by outputting a probability score in [0,1]. (b) Multi‐class DDI prediction classifies the specific interaction type $r_{ij}$, such as metabolic and synergistic, from the set of relations $R^{\text{DDI}}$. (c) Multi‐label DDI prediction identifies a subset of concurrent interaction types $Y\subseteq R^{\text{DDI}}$ for complex clinical scenarios, including simultaneous pharmacokinetics changes and pharmacodynamics effects. (d–f) Illustrate the tasks in DTI prediction. The model f processes a drug $e_{i}^{\text{drug}}$ and a target $e_{j}^{\text{target}}$. (d) Binary DTI prediction determines whether a drug can bind to the target protein. (e) drug–target affinity prediction is a regression task where f estimates the continuous binding strength values in R, which can be usually evaluated by $K_{d}$ and $IC_{50}$. (f) Mechanism of action prediction classifies the functional consequence of the binding, e.g., activation or inhibition, within the subset $R^{\text{MoA}}$.

The drug molecular interaction predictions are divided into DDI and DTI predictions. Both fields can be framed within a general relational learning context, operating on specific types of biomedical entities and relationships. Given a set E that consists of different entities, we define each entity in E as e. Meanwhile, we select R to be the set of all relationships r between each pair of entities. For both DDI prediction and DTI prediction, each metadata contains two entities, denoted as $e_{i}$ and $e_{j}\in E$, along with the relationship $r_{ij}\in R$ between $e_{i}$ and $e_{j}$. We illustrate the settings of all prediction tasks in Figure 1.

### 2.1 DDI Prediction Task

Let $D=\{e_{i}^{\text{drug}}\}$ be the set of all drug entities in E, and $R^{\text{DDI}}=\left\{r_{ij}^{\text{DDI}}\right\}\subset R$ be the set of all possible DDI interaction types. A known DDI is represented as a triplet $\left\langle e_{i}^{\text{drug}},r_{ij}^{\text{DDI}},e_{j}^{\text{drug}}\right\rangle$, where $e_{i}^{\text{drug}},e_{j}^{\text{drug}}\in D$ and $r_{ij}^{\text{DDI}}\in R^{\text{DDI}}$. The DDI prediction task can be formulated in several ways, most commonly as a multi‐class or binary prediction problem, and increasingly as a multi‐label learning problem.

#### 2.1.1 Binary DDI Prediction

This task aims to predict the existence of a certain drug–drug interaction. The goal is to build and learn a scoring function f:D×D→[0,1]. This function f takes two drug entities $e_{i}^{\text{drug}}$ and $e_{j}^{\text{drug}}$ in the defined triplet $\left\langle e_{i}^{\text{drug}},r_{ij}^{\text{DDI}},e_{j}^{\text{drug}}\right\rangle$ as input and outputs a probability score. A score of one indicates that a certain drug–drug interaction can take place between entities $e_{i}^{\text{drug}}$ and $e_{j}^{\text{drug}}$, while zero indicates no interactive effects would happen. This formulation answers the question of whether drug $e_{i}^{\text{drug}}$ and drug $e_{j}^{\text{drug}}$ interact with each other.

#### 2.1.2 Multi‐Class DDI Prediction

Based on binary DDI prediction, a further predictive scenario, called multi‐class prediction, is proposed. This task aims to predict the existence of the specific interaction type $r_{ij}$ given a pair of drugs $e_{i}^{\text{drug}}$ and $e_{j}^{\text{drug}}$. The goal is to learn a function $f:D\times D\to R^{\text{DDI}}$. In this context, the interaction type $r_{ij}^{\text{DDI}}$ can encompass a series of interaction categories, from high‐level pharmacological mechanisms to specific clinical adverse events. This setting answers the question of how drug $e_{i}^{\text{drug}}$ and drug $e_{j}^{\text{drug}}$ will interact.

#### 2.1.3 Multi‐Label DDI Prediction

In real‐world clinical scenarios, the interactions between two drugs are not solely limited to a single outcome; a pair of drugs may simultaneously exhibit multiple types of interactions, such as concurrent pharmacokinetics changes and pharmacodynamics adverse effects. This scenario is addressed by multi‐label DDI prediction. Different from the multi‐class setting that assumes a single mutually exclusive relation, this task aims to predict a subset of valid interaction types $Y\subseteq R^{\text{DDI}}$ for a given drug pair. The objective is to learn a mapping $f:D\times D\to {\left\{0,1\right\}}^{|R^{\text{DDI}}|}$, where the output is a binary vector indicating the presence or absence of each interaction type in $R^{\text{DDI}}$.

#### 2.1.4 Relationships Among Different DDI Prediction Tasks

The three formulations of DDI prediction tasks, i.e., binary, multi‐class, and multi‐label predictions, represent a progressive evolution path, which is moving from simple classification to deeper mechanistic profiling, and finally to real‐world clinical modeling. They differ in supervision signals, output structures, and biological interpretations, which in turn shape the principles of model design.

First, binary DDI prediction serves as a fundamental safety filter, providing a generalized risk evaluation on drug combination. The output structure is a simple scalar probability, and supervision relies mainly on positive and negative interaction pairs. To determine the presence of an interaction between two drugs, the primary design principle focuses on capturing the global topological relationship or overall structural similarity of the drug pair. Consequently, the prediction frameworks optimized via binary cross‐entropy are generally sufficient. Furthermore, to push the boundaries of binary prediction, these methods are trying advanced strategies such as contrastive learning and heterogeneous graph networks to achieve complex structural alignments and robust representations.

Second, multi‐class DDI prediction strengthens the biological interpretation by categorizing the specific mechanism of action or adverse drug event. Its output is a probability distribution over mutually exclusive classes. To distinguish between diverse interaction types, the proposed models should not only capture global information, but also explicitly reveal the local substructures or chemical motifs responsible for triggering different interactions. This functional demand drives the development of fine‐grained motif learning and relation‐aware architectures to capture distinct edge semantics. Meanwhile, addressing the long‐tailed distribution of rare but severe clinical events in multi‐class settings accelerates the development of specialized strategies such as meta‐learning or zero‐shot transfer.

Finally, multi‐label prediction further aligns with true clinical scenarios, where pharmacokinetic alterations and pharmacodynamic consequences can happen simultaneously. The prediction output is a high‐dimensional binary vector, supervised by non‐exclusive multi‐hot labels. This structural complexity introduces a major computational challenge: modeling the implicit dependencies and co‐occurrences between different labels. To capture these implicit inter‐label correlations and overcome data sparsity, the principles of model design focus on advanced learning paradigms, such as flow‐based message passing, automated subgraph selection, and utilization of LLMs.

### 2.2 DTI Prediction Task

DTI prediction can be described with a similar principle as DDI prediction. Concretely, let $T=\{e_{j}^{\text{target}}\}$ be the set of all protein target entities in E, and $R^{\text{DTI}}=\left\{r_{ij}^{\text{DTI}}\right\}\subset R$ be the set of all possible DTI interaction types. A known DTI is represented as a triplet $\left\langle e_{i}^{\text{drug}},r_{ij}^{\text{DTI}},e_{j}^{\text{target}}\right\rangle$, where $e_{i}^{\text{drug}}\in D$, $e_{j}^{\text{target}}\in T$, and $r_{ij}^{\text{DTI}}\in R^{\text{DTI}}$. While traditionally modeled as a binary classification problem, this domain has expanded to incorporate regression tasks for affinity and multi‐class tasks for mechanism predictions.

#### 2.2.1 Binary DTI Prediction

The binary classification is the most common setting in DTI prediction, aiming to predict the existence of an interaction between a drug and a target protein, i.e., learn a scoring function f:D×T→[0,1]. This function f takes a drug entity $e_{i}^{\text{drug}}$ and a target entity $e_{j}^{\text{target}}$ in the triplet $\left\langle e_{i}^{\text{drug}},r_{ij}^{\text{DTI}},e_{j}^{\text{target}}\right\rangle$ as input and outputs a probability score. A score of one indicates that an interaction can take place between entities $e_{i}^{\text{drug}}$ and $e_{j}^{\text{target}}$, while zero indicates no interactive effects would happen. This formulation answers the question of whether drug $e_{i}^{\text{drug}}$ binds to the target $e_{j}^{\text{target}}$.

#### 2.2.2 Drug–Target Affinity Prediction

Beyond identifying the binary existence of an interaction, measuring the strength of the binding is crucial for drug discovery. This task, known as Drug–Target Affinity (DTA) prediction, is formulated as a regression problem. The goal is to build and learn a function f:D×T→R. Here, the output corresponds to a continuous binding affinity value, which typically represents experimental metrics such as the dissociation constant ($K_{d}$), inhibition constant ($K_{i}$), or half‐maximal inhibitory concentration ($IC_{50}$), indicating how strongly drug $e_{i}^{\text{drug}}$ binds to target $e_{j}^{\text{target}}$. Furthermore, when this affinity prediction is scaled up to rank a massive library of candidate compounds against a specific target, it evolves into the critical drug discovery task of virtual screening.

#### 2.2.3 Mechanism of Action Prediction

To facilitate a deeper understanding of drug efficacy, it is necessary to determine the functional consequence of a drug binding to a target, such as whether it activates or inhibits the target. This task, referred to as Mechanism of Action (MoA) prediction, is similar to multi‐class prediction in the DDI domain. Let $R^{\text{MoA}}\subset R^{\text{DTI}}$ be the subset of relations representing functional mechanisms. The goal is to learn a function $f:D\times T\to R^{\text{MoA}}$. This task identifies the specific type of pharmacological effect a drug exerts on a target.

#### 2.2.4 Relationships Among Different DTI Prediction Tasks

Similar to DDI prediction, DTI prediction follows a hierarchical evolution from binary prediction to thermodynamic affinity, and finally to the functional mechanism of action. This progressive evolution in prediction settings further suggests the corresponding method choices.

First, binary DTI prediction also serves as the fundamental problem, identifying whether a binding event occurs. Its output is a simple scalar probability, supervised by discrete positive and negative drug pairs. In real‐world scenarios, this task faces noticeable data imbalance, sparsity of known interactions, and out‐of‐distribution (OOD) for novel molecules. To mitigate data scarcity and the over‐smoothing issues in deep neural networks, the adoption of self‐supervised contrastive learning, LLMs, and domain adaptation to enrich initial representations has emerged as a promising strategy. Furthermore, to capture complex heterogeneous topologies in binary screening, the integration of bidirectional attention mechanisms, multi‐view graph learning, and uncertainty quantification strategies is gradually becoming a necessity for binary DTI prediction.

Second, to capture the continuous spectrum of molecular binding, binding affinity prediction shifts the prediction paradigm from classification to regression. The output is a continuous scalar such as $IC_{50}$, $K_{d}$, or $K_{i}$ values, typically supervised and optimized via mean squared error. Compared with binary prediction, this continuous supervision requires models to learn fine‐grained physicochemical features rather than relying on broad topological relationships. These demands promote the design continuous optimization strategies and integrate multi‐modal features to accurately map multi‐source sequences and graphs to continuous binding strengths.

Beyond physical binding, MoA prediction addresses the functional consequences of the interactions. Even if a drug binds with a high affinity, its therapeutic value depends on its specific regulatory effect. Formulated as a multi‐class classification task and supervised by functional labels, accurate MoA prediction requires integrating deep structural features with downstream biological pathways. This functional complexity inspires the development of unified multi‐task learning frameworks that can effectively incorporate diverse interaction types to guarantee comprehensive and clinically reliable pharmacological profiling.

### 2.3 Summary

Both DDI and DTI prediction tasks share a common evolutionary trajectory, transitioning from basic topological associations to complex pharmacological mechanisms. Driven by an increasing number of biological entities and their relationships, AI technologies and deep learning methods should be continually developed to address the existing limitations in both fields. Concretely, building high‐quality and informative input features, designing effective, and insightful architectures, as well as developing novel learning paradigms are core perspectives for widening the boundaries of the current predictive models. Therefore, in the following section, we will systematically categorize and review the latest methods across these dimensions: feature engineering, core architectures, and learning paradigms. Meanwhile, we will also explore how these advanced methods meet the requirements for different DDI and DTI prediction tasks.

## 3 Advanced AI Technologies and Deep Learning Methods

**FIGURE 2:**
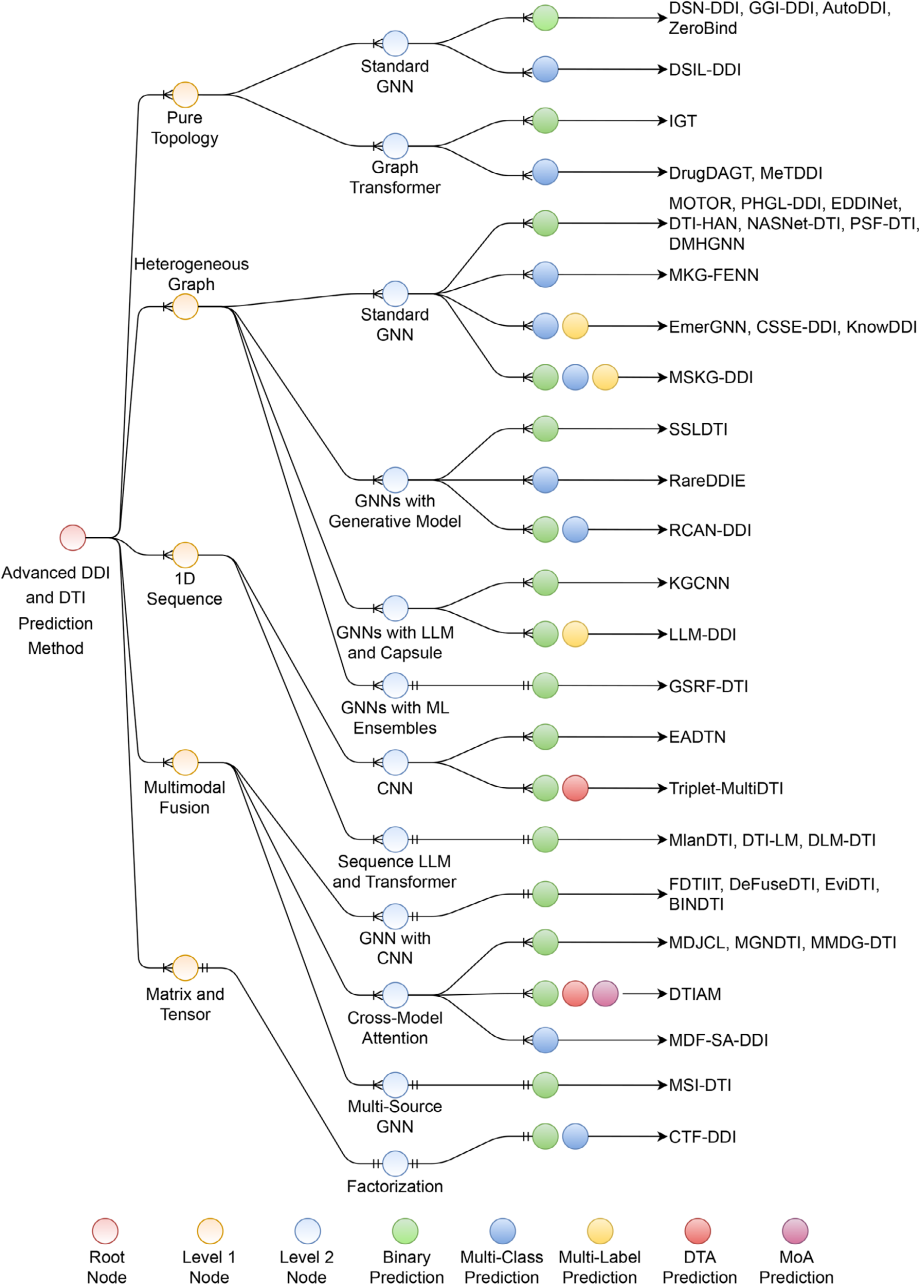
Unified taxonomy of advanced DDI and DTI prediction methods. The first layer (level 1 nodes colored orange) classifies methods by their input data modalities, encompassing pure topologies, heterogeneous graphs, 1D sequences, multimodal fusions, and tensor‐based similarity matrices. The second layer (level 2 nodes colored light blue) is organized by their core architectures, such as standard GNNs, Transformers, and CNNs. The terminal layer consisting of leaf nodes (distinguished by distinct dark colors) maps the models to their respective downstream computational tasks, including binary classification, multi‐class, multi‐label, drug–target affinity (DTA) prediction, mechanism of action (MoA) prediction, and virtual screening.

Driven by rapid algorithm advancements, predictive models in both fields, shown as Tables 1 and 2, have undergone a noticeable transformation. In this section, we systematically analyze such developments through three critical dimensions, feature engineering, core architecture, and contributions in learning paradigms, along with the tasks applicable to them defined in Figure 1. Meanwhile, to provide a clear structural overview, we construct a tree‐like taxonomy in Figure 2 to systematically categorize these methods. These perspectives trace the technical progression from initial naive frameworks to complex modeling systems that integrate heterogeneous knowledge and advanced mechanisms.

**TABLE 1:** Summary of the most recent state‐of‐the‐art DDI prediction models. The table categorizes each method by its core architecture, feature engineering methods, primary contribution, and task type.

Model	Core architecture	Feature engineering	Primary contribution
**Task Type: Binary**			
MOTOR [61] (2025)	GNN	Molecular Graph DDI Network	Multi‐granularity motif learning with iterative network topology refinement
PHGL‐DDI [57] (2025)	GNN	Molecular Graph DDI Network	Hierarchical graph learning combined with self‐supervised pre‐training
EDDINet [58] (2025)	GNN	Biological Networks	Multi‐graph contrastive learning based on information flow and consensus constraints
MDJCL [38] (2025)	GNN Transformer	Molecular SMILES Molecular Graph Molecular Conformation	Joint and cross‐learning of 1D/2D/3D multi‐dimensional molecular features
KGCNN [45] (2025)	GNN Capsule‐ Network	BKG	Introducing a spatial‐aware capsule network as a non‐linear aggregator
GGI‐DDI [60] (2024)	GNN	Molecular Graph	Identifying key substructures via granular computing for interpretability
AutoDDI [51] (2024)	GNN	Molecular Graph	Automated search for optimal GNN architecture to encode molecular graphs
DSN‐DDI [39] (2023)	GNN	Molecular Graph	Dual‐view learning of intra‐ and inter‐molecular atomic interactions
**Task Type: Multi‐Class**			
RareDDIE [55] (2025)	GNN VAE	Molecular Graph BKG	Dual‐granular structure‐driven variational representation for few‐shot and zero‐shot learning
MKG‐FENN [41] (2024)	GNN	Multimodal KGs	End‐to‐end fusion of four distinct knowledge graphs
MeTDDI [48] (2024)	Transformer	Molecular Graph Motif Graph	Motif‐based hierarchical self‐attention and emphasis on interpretability
DrugDAGT [47] (2024)	Graph‐ Transformer	Molecular Graph	Dual atom‐bond attention mechanism coupled with contrastive learning
DSIL‐DDI [56] (2023)	GNN	Molecular Graph	Domain‐invariant learning for generalizable and OOD prediction
MDF‐SA‐DDI [90] (2022)	CNN Autoencoder Transformer	Chemical Substructures Targets and Enzymes	Multi‐source drug fusion via diverse networks and latent feature fusion via transformer self‐attention
**Task Type: Multi‐Class & Multi‐Label**			
CSSE‐DDI [52] (2024)	GNN	DDI Network	Automated search for customized subgraph selection and encoding schemes
KnowDDI [42] (2024)	GNN	DDI Network BKG	Learning interpretable knowledge subgraphs to compensate for data sparsity
EmerGNN [43] (2023)	GNN	BKG DDI Network	Flow‐based GNN learning pairwise representations via biomedical paths for emerging drugs
**Task Type: Binary & Multi‐Class**			
RCAN‐DDI [50] (2025)	GNN GAN	Molecular Graph DDI Network	Relation‐aware structure learning fused with topology via cross‐adversarial network
CTF‐DDI [49] (2024)	Tensor‐ Factorization DNN	Similarity Matrices	Hessian and L2,1‐constrained tensor factorization for feature learning
**Task Type: Binary & Multi‐Label**			
LLM‐DDI [44] (2025)	GNN LLM	BKG Information Text	Leveraging LLM to generate initial entity embeddings
**Task Type: Binary, Multi‐Class & Multi‐Label**			
MSKG‐DDI [40] (2024)	GNN	Molecular Graph BKG	Fusing internal chemical structure with external biological knowledge

**TABLE 2:** Summary of the most recent state‐of‐the‐art DTI prediction models. The table categorizes each method by its core architecture, feature engineering methods, primary contribution, and task type.

Model	Core architecture	Feature engineering	Primary contribution
**Task Type: Binary**			
EviDTI [65] (2025)	GNN CNN	Molecular Graph Molecular Conformation Protein Sequence	Evidential deep learning for uncertainty quantification and multimodal fusion
NASNet‐DTI [76] (2025)	GNN	Heterogeneous Network	Node dependent local smoothing strategy to address over‐smoothing
MMDG‐DTI [80] (2025)	LLM GNN	Multi‐domain Datasets	Hybrid fusion of LLM and GNN with domain adversarial training
PSF‐DTI [85] (2025)	GNN	DTI Network	Multi‐graph fusion with pseudo‐label supervision
BINDTI [72] (2025)	GNN CNN	Molecular Graph Protein Sequence	Bi‐directional intention network fusion with ACmix encoder
DMHGNN [68] (2025)	GNN	Heterogeneous Network	Multi‐view graph learning mechanism combining meta‐paths and similarity
MlanDTI [81] (2024)	LLM Transformer	Protein Sequence Molecular SMILES	Multilevel attention network with semi‐supervised domain adaptation
MSI‐DTI [89] (2024)	GNN	Multi‐source Features	Fusion of diverse features via multi‐head self‐attention
DTI‐HAN [83] (2024)	GNN	Heterogeneous Network	Heterogeneous graph attention network with fuzzy classification to reduce false negatives
FDTIIT [74] (2024)	GNN CNN	Molecular Graph Protein Sequence	Interactive information extraction via mutual attention and HSIC for dependency trade‐off
SSLDTI [79] (2024)	GNN Autoencoder	Heterogeneous Network	Self‐supervised learning with local subgraph and global influence contrastive tasks
DeFuseDTI [77] (2024)	CNN GNN	Protein Sequence Molecular Graph	Dual‐branch encoder and multiview fusion attention for interpretability
DTI‐LM [70] (2024)	LLM GNN	Protein Sequence Molecular SMILES	Leveraging frozen LLM embeddings with GAT for cold‐start scenarios
MGNDTI [67] (2024)	GNN Transformer	Molecular SMILES Molecular Graph Protein Sequence	Multimodal drug representation fused via a gating mechanism
EADTN [64] (2024)	CNN	Protein Sequence Molecular SMILES	Ensemble framework with feature adaptation and fine‐tuning enhanced by clustering strategy
GSRF‐DTI [69] (2024)	GNN Random‐ Forest	Heterogeneous Network	Construction of drug–target pair network processed by GraphSAGE and random forest
DLM‐DTI [91] (2024)	LLM Transformer	Molecular SMILES Protein Sequence	Hint‐based knowledge adaptation with teacher‐student architecture for a lightweight target encoder
ZeroBind [82] (2023)	GNN	Molecular Graph Protein Graph	Zero‐shot prediction via subgraph matching and information bottleneck
IGT [71] (2022)	Graph‐ Transformer	Molecular Graph Protein Graph	Modeling intermolecular spatial dependencies via graph Transformer
**Task Type: Binary & DTA**			
Triplet‐ MultiDTI [63] (2023)	CNN	Protein Sequence Molecular SMILES	Representation learning optimized by triplet loss for data fusion
**Task Type: Binary, DTA & MoA**			
DTIAM [78] (2025)	Transformer	Molecular Graph Protein Sequence	Unified framework with self‐supervised pre‐training on label‐free data

### 3.1 AI‐Driven DDI Prediction

#### 3.1.1 Method Evolution in DDI Prediction

The deep learning models in these fields employ various strategies to construct and process input features, ranging from intrinsic molecular structures to external semantic knowledge. In terms of intrinsic feature mining, MDJCL [38] maximizes produced information by fusing 1D sequences, 2D graphs, and 3D conformational structures to capture physicochemical properties at multiple granularities. Similarly, DSN‐DDI [39] treats the drug pair as a unified view, simultaneously learning intra‐molecular atomic features and inter‐molecular interaction patterns. Expanding beyond solely molecular features, MSKG‐DDI [40], and MKG‐FENN [41] integrate heterogeneous BKGs to provide broader biological contexts. KnowDDI [42] and EmerGNN [43] refine this approach by extracting relation‐aware evidence subgraphs rather than using the entire graph, effectively compensating for sparse interaction labels. An important shift is observed in LLM‐DDI [44], which moves away from structural inputs to leverage LLMs. By processing textual descriptions of drugs, it generates semantic embeddings that capture rich biomedical concepts often implicit in structured databases.

In terms of the model architecture, it has evolved to address specific structural limitations and capture complicated dependencies. KGCNN [45] improves the standard message passing by introducing spatial‐aware capsule networks, preserving the hierarchical poses and spatial relationships among molecular substructures. To capture global dependencies often missed by local GNNs, TIGER [46] injects topological information directly into a dual‐channel heterogeneous graph Transformer. This trend is further strengthened by DrugDAGT [47] and MeTDDI [48], which utilize specialized attention mechanisms to model atom‐bond interactions and functional motifs, respectively. Different from deep neural networks, CTF‐DDI [49] demonstrates the efficacy of rigorous mathematical modeling via constrained tensor factorization reinforced by Hessian regularization. Meanwhile, RCAN‐DDI [50] repurposes GANs for feature fusion, enforcing the complementarity between structural and topological representations.

The evolution in learning paradigms primarily arises from human bias, data scarcity, and interpretability. To reduce reliance on manual model design, AutoDDI [51], and CSSE‐DDI [52] apply neural architecture search (NAS) [53, 54] to automate the selection of GNN encoders and subgraph sampling schemes. Regarding the cold‐start and generalizability challenges, RareDDIE [55], and DSIL‐DDI [56] leverage meta learning for rare events and domain‐invariant substructure learning for generalizability improvement. To leverage unlabeled data, PHGL‐DDI [57] and EDDINet [58] adopt self‐supervised contrastive learning strategies to extract robust representations from molecular graphs and biological networks. Finally, DDI‐GCN [59] and GGI‐DDI [60] enhance interpretability by identifying key chemical motifs responsible for interactions, while MOTOR [61] employs iterative topology refinement to dynamically correct noisy network structures.

#### 3.1.2 Method‐Task Alignment in DDI Prediction

We then map the latest computational methods back to the distinct DDI task formulations and design principles established in Section 2.1.4. As analyzed, for binary DDI prediction, the primary requirement is to identify interaction existence based on global topological extraction. To achieve this, models employing standard graph aggregators or tensor factorization [38, 49, 51] are highly suitable. Moreover, to further fulfill the requirement for robust binary classification through drug structural alignments, novel strategies such as dual‐view learning [39], granular computing [60], motif‐oriented learning [61], spatial‐aware capsule networks [45], and various advanced graph networks [46, 57, 58, 62] have been effectively adopted as structural enhancements.

Transitioning to multi‐class DDI prediction, model design principles shift toward distinguishing specific pharmacological mechanisms. Aligning with the necessity to extract fine‐grained features and local motifs rather than relying solely on macroscopic topologies, novel solutions have been proposed. These include customized subgraph selection [52], multi‐scale motif extraction [40], and architectures with knowledge embedding [41, 42, 48, 50], which are specifically designed for the ability to explicitly isolate functional groups. Additionally, to satisfy the requirement for capturing complex edge semantics, domain‐invariant learning [56] and dual‐attention contrastive learning [47] have been introduced. Furthermore, to address the data sparsity of rare adverse events in multi‐class settings, meta‐learning strategies have proven highly effective [55].

Finally, addressing the clinical complexity in multi‐label scenarios requires models to process implicit dependencies among co‐occurring labels. To address these specific challenges, the field has progressed toward effective learning strategies. Frameworks integrating flow‐based graph networks [43] and large language models [44] represent the insightful choices here, as they provide proven capacities to handle imbalanced distributions and uncover high‐dimensional label correlations.

### 3.2 AI‐Driven DTI Prediction

#### 3.2.1 Method Evolution in DTI Prediction

Similiar to DDI prediction, the developments of DTI prediction in data engineering have shifted from simple sequence inputs to higher‐dimensional and multimodal representations. While models like TripletMultiDTI [63] and EADTN [64] continue to refine feature adaptation on standard 1D sequences and SMILES, EviDTI [65] breaks the barrier by explicitly incorporating 3D molecular conformations via GeoGNN [66], acknowledging spatial structure as a prerequisite for binding. Multimodal integration involves fusing diverse views. For instance, MGNDTI [67] combines molecular graphs, SMILES strings, and protein sequences through a gating mechanism to regulate feature importance. In the context of heterogeneous networks, DMHGNN [68] employs a multi‐view learning mechanism that synthesizes meta‐paths with similarity networks, while GSRF‐DTI [69] constructs a specialized drug–target pair network to capture edge‐level associations explicitly. Furthermore, the field is leveraging pre‐trained foundation models over model training from scratch. DTI‐LM [70] utilizes frozen embeddings from large language models, allowing graph attention networks to reason over high‐quality semantic features even in cold‐start scenarios.

The model architectural innovations focus on capturing long‐range dependencies and refining interaction modeling. IGT [71] adapts the Transformer encoder for molecular graphs, while BINDTI [72] introduces a bi‐directional intention network fused with an ACmix encoder to capture complex interaction between drugs and targets. Addressing the redundancy in high‐dimensional features, ReduMixDTI [73] employs a reconstruction‐based reduction mechanism coupled with interpretable multi‐channel cross‐attention. Similarly, FDTIIT [74] utilizes Hilbert‐Schmidt independence criterion to trade off between interactive and independent information extraction. In the realm of graph neural networks, GENNDTI [75] introduces biologically meaningful router nodes to facilitate efficient information exchange through the graph. To mitigate the over‐smoothing problem in deep GNNs, NASNet‐DTI [76] implements a node‐dependent local smoothing strategy, preserving the distinctiveness of node representations during message propagation. DeFuseDTI [77] enhances interpretability through a dual‐branch encoder system that uses multi‐view fusion attention to highlight critical binding factors.

The novel model learning paradigms have been developed to address label scarcity, distribution shifts, and prediction reliability. To tackle the lack of labeled data, DTIAM [78] establishes a unified framework based on self‐supervised pre‐training, learning robust representations from vast label‐free datasets. SSLDTI [79] further refines this scheme by employing contrastive learning tasks that maximizes the mutual information between local subgraphs and global model structures. As for the distribution shift between training and real‐world data, MMDG‐DTI [80], MlanDTI [81] and ZeroBind [82] incorporate domain generalizability and meta learning techniques. They use domain adversarial training or apply semi‐supervised domain adaptation to generalize to out‐of‐distribution samples. To reduce the impact of potential false negatives in unlabeled data, DTI‐HAN [83] designs a fuzzy classification mechanism [84] combined with graph attention. Finally, PSF‐DTI [85] leverages pseudo‐label supervision to guide multi‐graph fusion, while EviDTI [65] introduces evidential deep learning [86, 87] to quantify prediction uncertainty, providing a reliability metric alongside the binding score.

#### 3.2.2 Method‐Task Alignment in DTI Prediction

As described in Section 2.2.4, for binary DTI screening, models frequently face the challenges of OOD molecules and data sparsity in cold‐start scenarios. To address these data limitations, several paradigms have emerged as promising solutions, including self‐supervised contrastive learning [79, 88], zero‐shot subgraph matching [82], and supervised multi‐graph fusion with pseudo labels [85]. To mitigate domain shifts and augment representations, leveraging LLMs and domain adaptation [70, 80, 81] has become a favored architectural choice. To perform spatial drug–target alignments and capture complex interaction topologies in binary tasks, bidirectional attention mechanisms [72, 77], multimodal gating networks [67, 89], interactive information extraction [74], and heterogeneous graph networks [68, 76, 83] are effectively utilized. Furthermore, given the noise in interaction networks, evidential deep learning [65] and robust frameworks [64, 69] are integrated to quantify uncertainty and reduce false negatives.

As the prediction setting transfers to binding affinity, the requirement shifts toward capturing the thermodynamic properties. This regression demand directs architectural choices toward continuous optimization strategies and multi‐modal feature integration. Frameworks employing representation learning optimized by triplet loss [63] and self‐supervised pre‐trained transformers [78] represent optimal candidates for this task, as they accurately map multi‐source sequences and molecular graphs to continuous binding strengths rather than relying on broad topological proximity.

Finally, predicting the functional mechanism of action demands the integration of physical binding with downstream biological pathways. For this highly complex multi‐class setting, unified multi‐task frameworks [78] represent a powerful method class. By incorporating multi‐view biological contexts and self‐supervised pre‐training, such frameworks guarantee clinically reliable interaction profiling with the goal of real‐world clinical modeling.

## 4 Shared Patterns and Trends

The parallel evolution of DDI and DTI predictions highlights a convergence in patterns of feature engineering and trends of model design.

### 4.1 From Feature Extraction to Knowledge Integration

First, a major shift observed across both domains is the move from simple feature extraction to holistic knowledge integration. Previous models often relied on the assumption that drugs and targets could be defined solely by their intrinsic chemical or sequence features. However, recent methods have largely adopted a systemic perspective, placing biological entities within more comprehensive BKGs. This evolution reflects a consensus that biological identity of a given biology molecule is defined not only by its structure, but also by its extrinsic interaction landscape, including pathways, enzymes, and diseases. By encoding these heterogeneous relationships, models like KnowDDI [42], and EmerGNN [43] move from simple pattern matching to reasoning about the underlying biological mechanisms, thereby capturing the rich context necessary to predict interactions for entities with sparse direct evidence that structure‐based models often miss.

### 4.2 The Adoption of Pre‐Trained Foundation Models

Second, both fields are moving away from training models from scratch to a pre‐train and fine‐tune paradigm. The use of foundation models, particularly LLMs trained on vast protein sequences and chemical notations, represents an improvement in feature engineering. Unlike previous deep learning models that need to learn biochemical rules from limited labeled data, these models, such as LLM‐DDI [44] and DTI‐LM [70], incorporate fundamental biochemical knowledge into downstream tasks. By leveraging representations learned from the entirety of known chemical and biological space, the data scarcity issue could be effectively mitigated, providing robust, generalizable initial embeddings that capture essential physicochemical properties even for novel compounds under a cold‐start scenario.

### 4.3 Integration of GNNs and Transformer Architectures

Third, the model architecture is shifting toward a robust integration of GNNs and Transformers, rather than relying solely on either architecture [92]. GNN is capable of encoding the topological structure of molecules and interactions among them. Despite its limitations in capturing long‐range dependencies, incorporating the self‐attention mechanism from Transformer allows models to capture global context simultaneously. Consequently, the hybrid deep learning framework that combines the advantages from both sides creates a more powerful and unified backbone for complicated interaction prediction.

### 4.4 The Demand for Trustworthy AI

As deep learning models play a critical role in drug discovery, the “black box” nature of the traditional model predictions is becoming insufficient to provide biological insights and guide drug design [93, 94]. There is a growing imperative for these models to be transparent and reliable. Consequently, both fields are integrating interpretable mechanisms directly into model architectures, moving beyond post‐hoc analysis to intrinsic explainability, such as reasoning based on specific chemical motifs or attention maps that identifies key binding residues [48, 77]. Concurrently, uncertainty quantification methods such as evidential deep learning are gaining more attention. This ensures models can quantify their uncertainty, facilitating the distinction between high‐confidence predictions and uncertain guesses in clinical scenarios.

Furthermore, in addition to designing transparent models with increased interpretability, the reliability in clinical and pharmacological applications should be rigorously validated by biological evidence [95]. Consequently, incorporating biological validation strategies is becoming a critical standard for evaluating DDI and DTI prediction models. At the molecular and cellular levels, in vitro assays serve as the key standard. For DDI predictions, cell viability assays can directly confirm synergistic or antagonistic toxicities between a pair of drugs [96]. For DTI predictions, surface plasmon resonance [97] and isothermal titration calorimetry [98] are widely used to quantify binding affinities. While wet‐lab validations are constrained by cost and time, physics‐based approaches, such as molecular dynamics simulations [99, 100, 101] serve as an effective biophysical bridge to verify the structural stability and thermodynamic feasibility of the AI‐predicted interactions. Finally, at the population level, real‐world data provides necessary clinical validations. By comparing computational predictions with the FDA adverse event reporting system [102], researchers can determine whether the identified drug interactions and their associated adverse events occur in patients. These technological strategies ensure the translational value of AI models from theoretical computation to real‐world applications.

## 5 Common Limitations and Challenges

### 5.1 Insufficient Generalizability

**FIGURE 3:**
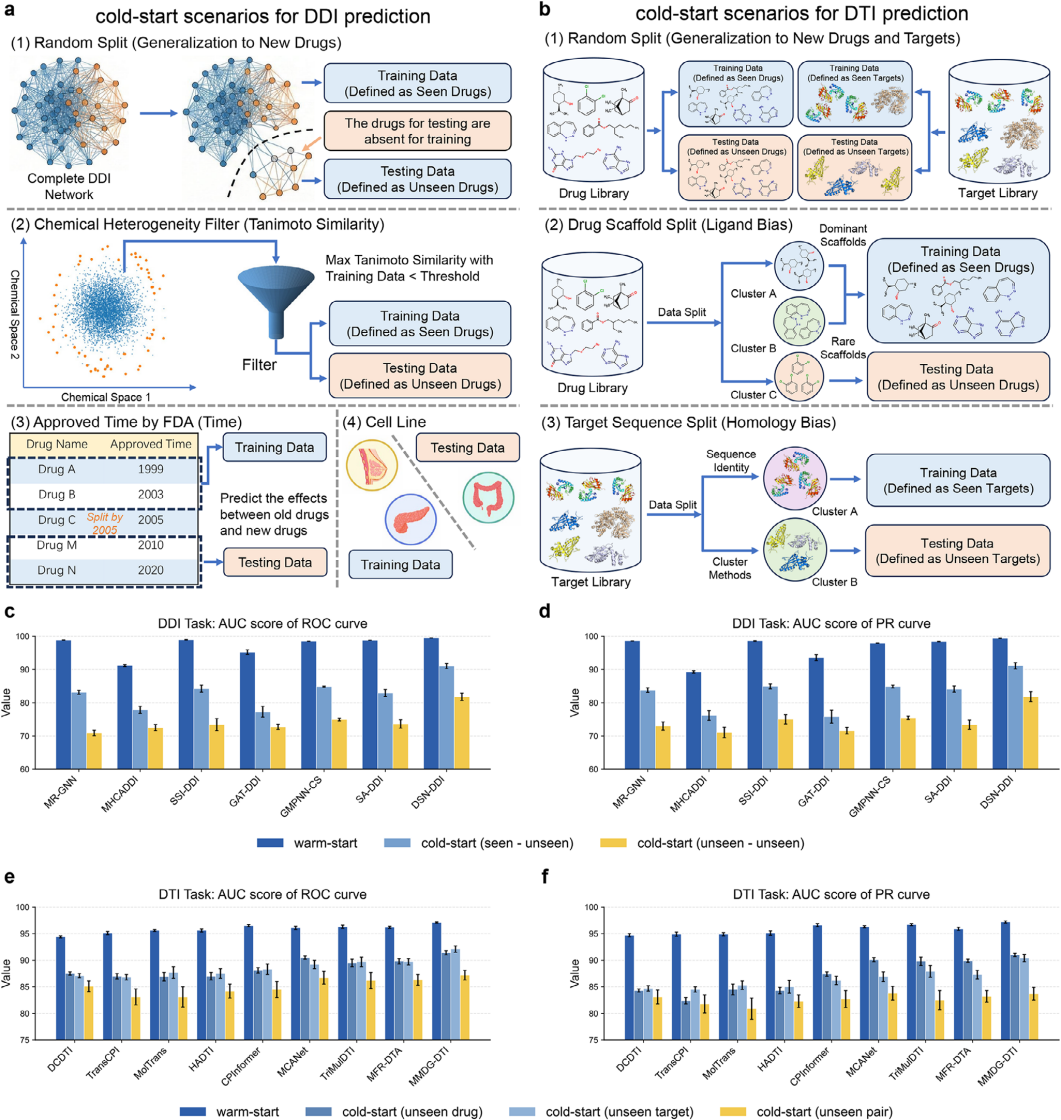
Illustrations on different kinds of cold‐start scenarios where at least one entity (either drug or target) in the test set is unseen during training and performance evaluations between cold‐start and warm‐start scenarios for both DDI and DTI predictions. (a) For DDI prediction, the cold‐start scenarios are designed by dataset split strategies including the random split and field‐specific splits, e.g., chemical heterogeneity filtering based on Tanimoto similarity, the drug approved dates by FDA, and cell line split. These data split strategies mimic the real‐world scenarios where models should accurately predict interactions for completely novel therapeutic agents. (b) For DTI prediction, the cold‐start scenarios are designed by dataset split strategies including the random split and field‐specific splits, e.g., drug scaffold split and target sequence identity filtering to examine the generalizability ability across unseen chemical scaffolds and protein sequences. (c, d) Performance comparison between cold‐start scenario designed by the random split and warm‐start scenario on DrugBank dataset for DDI prediction. The area under curve (AUC) scores for both ROC curves and PR curves are evaluated. (e, f), Performance comparison between cold‐start scenario designed by the random split and warm‐start scenario on BindingDB dataset for DTI prediction. The area under curve (AUC) scores for both ROC curves and PR curves are evaluated.

A critical challenge existing in both DDI and DTI prediction fields is the models' generalizability to novel chemical spaces. To examine this capacity, we stratify the prediction tasks into two categories: cold‐start scenarios, where at least one entity (either drug or target) in the test set is unseen during training; and warm‐start scenarios, where both the drug and target entities in the test set have been observed in the training set. As shown in Figure 3a,b, the cold‐start scenarios are designed by dataset split strategies. The random split strategy randomly partitions entities into training and testing sets, applied to both DDI and DTI predictions, while the two fields also employ specific split strategies, respectively. For DDI prediction, filtering chemical overlaps using Tanimoto similarity thresholds ensures the testing process on truly novel chemical spaces. Similarly, the temporal splitting strategy based on FDA approval dates emulates the chronological nature of pharmaceutical development. In addition, for drug–drug synergy prediction involving context‐dependent responses, e.g., DrugComb [103, 104], cell line‐based splitting is employed to evaluate whether models can generalize learned synergistic patterns to unseen biological environments. For DTI prediction, dividing training and test sets based on Bemis‐Murcko scaffold clustering or protein sequence identity could be adopted, enabling models to learn transferable interaction patterns across distinct chemical spaces and protein families. Collectively, these cold‐start designs are critical for preventing information leakages and demonstrate the capability to extrapolate to a broader range of chemical entities.

While advanced deep learning models exhibit remarkable prediction accuracy under warm‐start scenarios, when evaluated on several datasets, such as DrugBank [105, 106], PDBbind [107, 108], and BIOSNAP [109, 110], the distinct performance drops are often observed under the cold‐start scenarios in the same dataset. To examine this phenomenon, we analyzed and compared the model prediction performance under cold‐start scenario designed by general random split strategy and that under warm‐start scenario. As shown in Figure 3c–f, for both DDI and DTI predictions, the AUC score of ROC and PR curves dropped in the cold‐start scenarios by a large margin. Specifically, when both entities of a pair are unseen in the training set, the models performed much worse compared with the scenarios where only one entity is unseen. Therefore, although deep learning models perform well for existing drugs and targets, the insufficient generalizability is a crucial challenge for new drug design.

**FIGURE 4:**
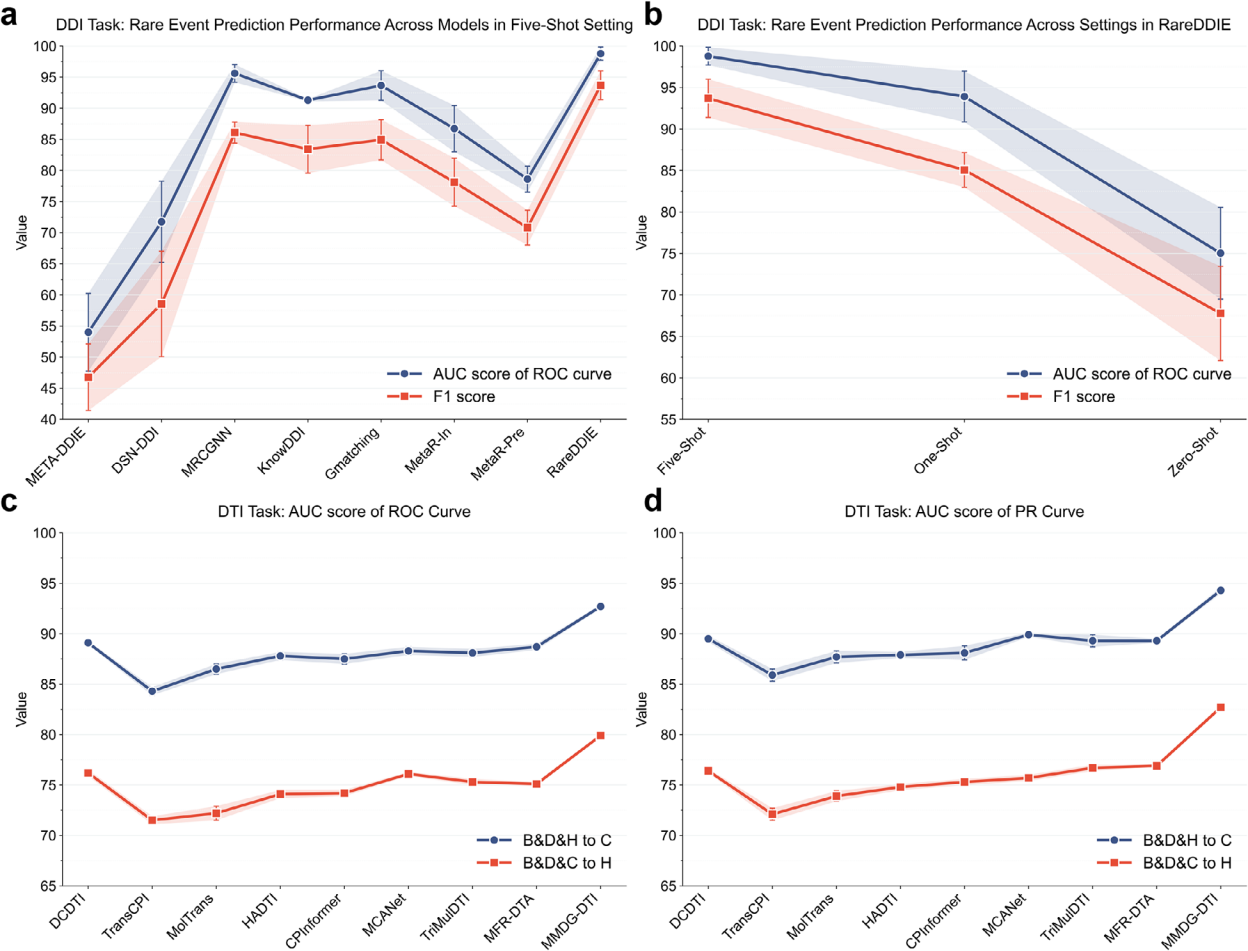
Performance evaluation in few‐shot, zero‐shot, and transfer learning scenarios for DDI and DTI predictions. (a) Performance evaluation of various DDI prediction models in the few‐shot (five‐shot) learning scenario. The five‐shot setting entails providing the model with five relevant drug‐pair examples for each test instance. (b) Performance evaluation the DDI prediction model RareDDIE in five‐shot, one‐shot, and zero‐shot scenarios. With less information provided for retrieval from few‐shot to zero‐shot scenarios, the model's performance shows noticeable drops. In (a) and (b), rare event prediction refers to the prediction for drug‐pair event types with fewer than 20 samples. The original results were derived from RareDDIE [55] and then re‐analyzed in this work. (c) Performance evaluation of various DTI prediction models in transfer learning scenario, measured by the area under the ROC curve (AUC score of ROC curve). (d) Performance evaluation of various DTI prediction models in transfer learning scenario, measured by the area under the PR curve (AUC score of PR curve). In (c) and (d), four datasets are selected for model training and inference, i.e., BindingDB (B), DrugBank (D), Human (H), and C.elegans (C). The term B&D&H to C means that MMDG‐DTI is trained on the BindingDB (B), DrugBank (D), and Human (H) datasets as multiple source domains, and is subsequently evaluated on the C.elegans (C) dataset as the unseen target domain to examine its cross‐domain transferability and generalization capabilities. Similar protocols are also applied for the settings B&D&C to H. The original results were derived from MMDG‐DTI [80] and then re‐analyzed in this work. In (a) to (d) the standard deviations in each experiment are shown with shadow.

To further evaluate this challenge in DDI and DTI predictions, we analyzed various models' performance in few‐shot learning, zero‐shot learning, and transfer learning scenarios for both predictions. First, to evaluate how models deal with data sparsity, we reanalyzed the performance of multiple baseline architectures under few‐shot and zero‐shot learning scenarios reported by RareDDIE [55], as shown in Figure 4a,b. In this analysis, we specifically focused on rare DDI event prediction, referring to the prediction for drug‐pair event types with fewer than 20 samples. Figure 4a compares the AUC scores of ROC curves of various models in the five‐shot scenario, where the model is provided with only five relevant drug‐pair examples for each test instance. This setting rigorously evaluated whether these well‐trained models can leverage minimal prior examples to produce accurate predictions for rare events. Furthermore, taking RareDDIE as an example, Figure 4b examines model's performance in five‐shot, one‐shot, and zero‐shot scenarios. The distinctly declined performance on both AUC and F1 scores from five‐shot to zero‐shot settings indicates that data sparsity remains a critical bottleneck for model generalization, particularly when predicting interactions for novel entities. These findings further strengthen the necessity for continued architectural and strategic innovations to better extract and share transferable knowledge for accurate interaction predictions.

Importantly, few studies incorporate meta‐learning and transfer learning into drug interaction prediction models. Even among such preliminary attempts, generalization across heterogeneous biomedical domains remains imbalanced. As shown in Figure 4c,d, when evaluating MMDG‐DTI [80] in the cross‐dataset transfer learning scenario, the AUC score of PR curve dropped to approximately 0.70. This decline reveals a critical challenge: how to improve model's generalizability against domain shifts. Therefore, future research should focus on how domain adaptation is conducted. Developing effective strategies to better align feature distributions across diverse datasets will be essential to ensure that the transferred knowledge remains robust and useful across varying biomedical contexts.

### 5.2 Shortcut Learning

Another noticeable challenge is the shortcut learning phenomenon in deep learning models [114, 115, 116, 117, 118]. Rather than learning fundamental physical interaction laws during the training process, models tend to leverage data biases and spurious correlations within training samples, such as memorizing dominant ligand substructures, specific assay conditions or entity frequencies.

To disentangle genuine interaction signals from entity‐level memorization, rigorous validation protocols are indispensable. A representative approach is the label‐reversal experiment performed by TransformerCPI [119]. This study found that models could achieve misleadingly high performance on conventional datasets by simply memorizing the occurrence frequencies of specific ligands rather than learning the actual binding mechanisms. To evaluate the potential bias, a well‐designed symmetric evaluation dataset was constructed: if drug $e_{i}^{\text{drug}}$ interacts with $e_{m}^{\text{target}}$ but not with $e_{n}^{\text{target}}$, the dataset ensures there is another pair that drug $e_{j}^{\text{drug}}$ interacts with target $e_{n}^{\text{target}}$ but not with $e_{m}^{\text{target}}$. By compelling the model to yield opposite predictions for the same entity under different contexts, this paired‐control design explicitly examines whether a model captures the true pairing logic rather than relying on dataset biases. The label‐reversal experiments serve as a reliable standard for evaluating shortcut learning problems in binary interaction tasks.

To further investigate this issue, we propose the self‐paired pseudo‐negative experiment. The experimental protocol was designed to decouple real molecular interaction learning from entity‐level memorization. First, we quantified the positive label rate for each unique drug or target across the entire training dataset, serving as a metric for entity popularity. Subsequently, after the model training convergences, we predicted the interaction probability by taking one entity itself as input, i.e., $\left\langle e_{i}^{\text{drug}},e_{i}^{\text{drug}}\right\rangle$ for DDI prediction (DDI‐drug) and $\left\langle e_{i}^{\text{drug}},e_{i}^{\text{drug}}\right\rangle$ (DTI‐drug) and $\left\langle e_{i}^{\text{target}},e_{i}^{\text{target}}\right\rangle$ (DTI–target) for DTI prediction. These entity self‐pairs serve as pseudo‐negative samples. Theoretically, the interaction probability of these samples should be zero as the target or drug itself distinctly neither binds to itself nor possess a side or synergistic effect.

**FIGURE 5:**
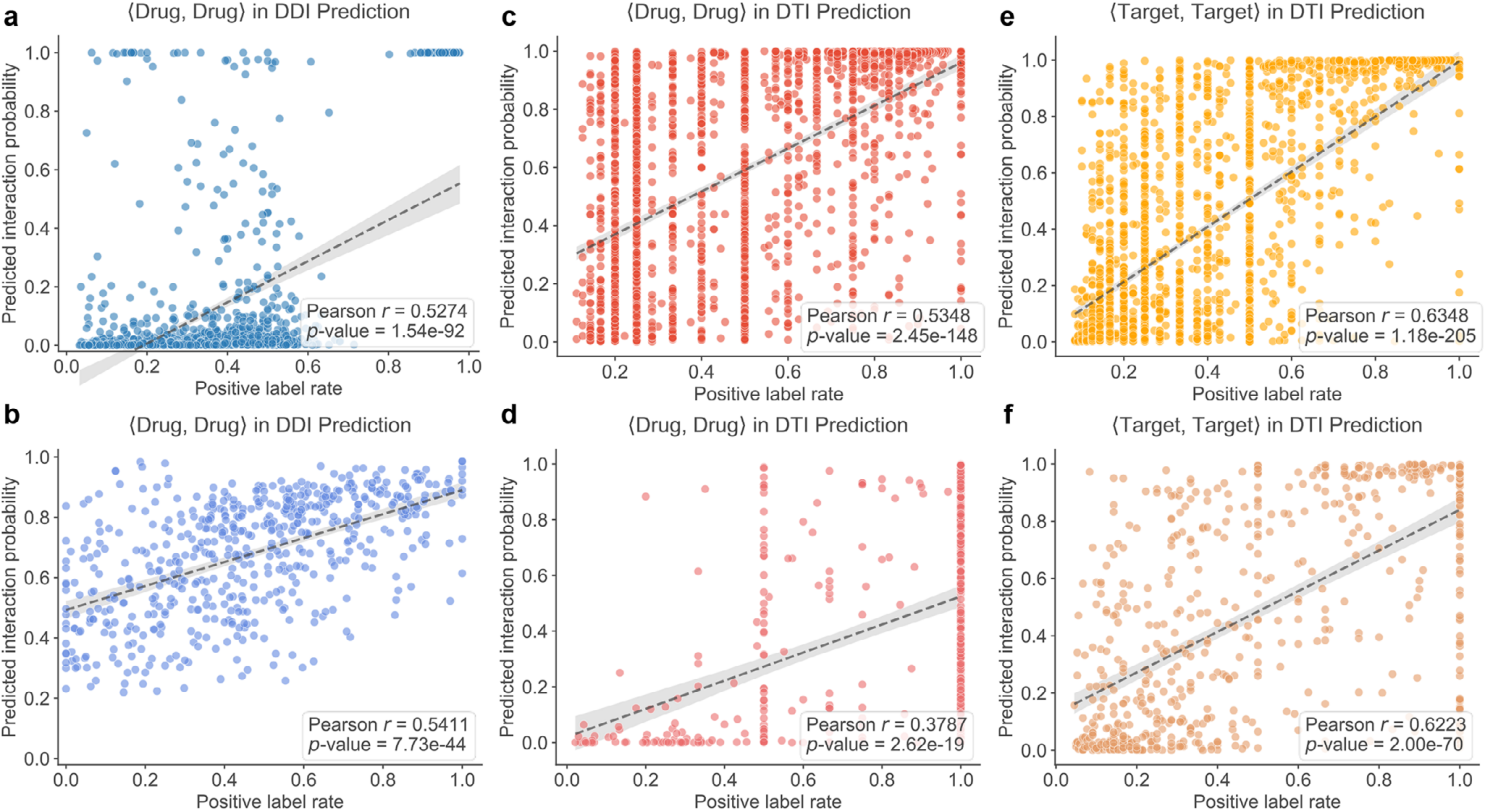
Correlation analysis between the positive label rate of an entity in the training set and its predicted interaction probability when provided as the sole input. The positive label rate is defined as the ratio of positive samples to total samples for each entity (drug or target) in the training set. (a) The predicted interaction probability when a drug provided as the sole input (termed as “⟨Drug, Drug⟩”) for DDI prediction is calculated by MDJCL [38] (fuses sequential features encoded by Transformers and spatial features extracted by GNNs) on Pang's dataset [111]. (b) The predicted interaction probability when a drug provided as the sole input (termed as “⟨Drug, Drug⟩”) for DDI prediction is calculated by MDF‐SA‐DDI [90] (selects CNNs and Autoencoders to capture sequential features from similarity spectrum, and then fuses them via Transformers) on Deng's dataset [112]. (c) The predicted interaction probability when a drug provided as the sole input (termed as “⟨Drug, Drug⟩”) for DTI prediction is calculated by MGNDTI [67] (fuses sequential features encoded by Transformers and spatial features extracted by GNNs) on BioSNAP dataset [109, 110]. (d) The predicted interaction probability when a target provided as the sole input (termed as “⟨Target, Target⟩”) for DTI prediction is calculated by MGNDTI [67] (fuses sequential features encoded by Transformers and spatial features extracted by GNNs) on BioSNAP dataset [109, 110]. (e) The predicted interaction probability when a drug provided as the sole input (termed as “⟨Drug, Drug⟩”) for DTI prediction is calculated by DLM‐DTI [91] (employs pre‐trained LLMs to encode sequences and performs distillation via a light‐weight Transformer) on BindingDB dataset [113]. (f) The predicted interaction probability when a target provided as the sole input (termed as “⟨Target, Target⟩”) for DTI prediction is calculated by DLM‐DTI [91] (employs pre‐trained LLMs to encode sequences and performs distillation via a light‐weight Transformer) on BindingDB dataset [113]. In (a) to (f), the Pearson correlation coefficients are calculated, and two‐sided Student's t‐test are performed.

To cover more DDI and DTI prediction models with different core architectures, we selected MDJCL [38], MGNDTI [67], MDF‐SA‐DDI [90] and DLM‐DTI [70] for self‐paired pseudo‐negative experiments. These models possess a wide range of model architectures, including Transformers, GNNs, CNNs, Autoencoders, and LLMs, enabling a comprehensive assessment on whether shortcut learning is a pervasive issue across different architectures. Concretely, MDJCL [38] extracts sequential and structural features using Transformers and GNNs, respectively, integrating them via a hybrid cross‐attention module. MDF‐SA‐DDI [90] processes different drug pairs through parallel branches, including CNNs and Autoencoders followed by the aggregation of the extracted latent vectors via a Transformer‐based module. MGNDTI [67] relies on Transformers and GNNs to encode sequential and structural information, aligning drug and target representations through a multimodal gating network. In addition, DLM‐DTI [91] employs pre‐trained LLMs to encode molecular sequences, refining the target features via a teacher‐student distillation to perform predictions. As shown in Figure 5, a large portion of samples exhibit high interaction probabilities, indicating models fail to identify such pseudo‐negative samples. We then calculated the Pearson correlation between the predicted interaction probability and the positive label rates of these entities. The analysis reveals a significant statistical dependency with Pearson correlations ranging from 0.3787 to 0.6348, and p‐values consistently less than $10^{-19}$. These results indicate that current models tend to memorize popular biological entities, assigning high interaction probabilities based on data prevalence rather than capturing true molecular interaction mechanisms.

Complementary to the label‐reversal experiment, the proposed self‐paired pseudo‐negative analysis provides a more scalable evaluation strategy. Specifically, different from the label‐reversal experiment that requires the manual construction of strict symmetric pairs, the correlation analysis on pseudo‐negative samples can quantify the continuous impact of degree bias across the entire dataset. Furthermore, the self‐paired design can be further applied to multi‐class and multi‐label DDI prediction tasks, where interactions are not only binary, but also involve complex, co‐occurring semantic relations. Taken together, these two validation protocols offer complementary perspectives for detecting shortcut learning issues in drug interaction modeling.

### 5.3 Heterogeneity in Data Quality and Curation Standards

**FIGURE 6:**
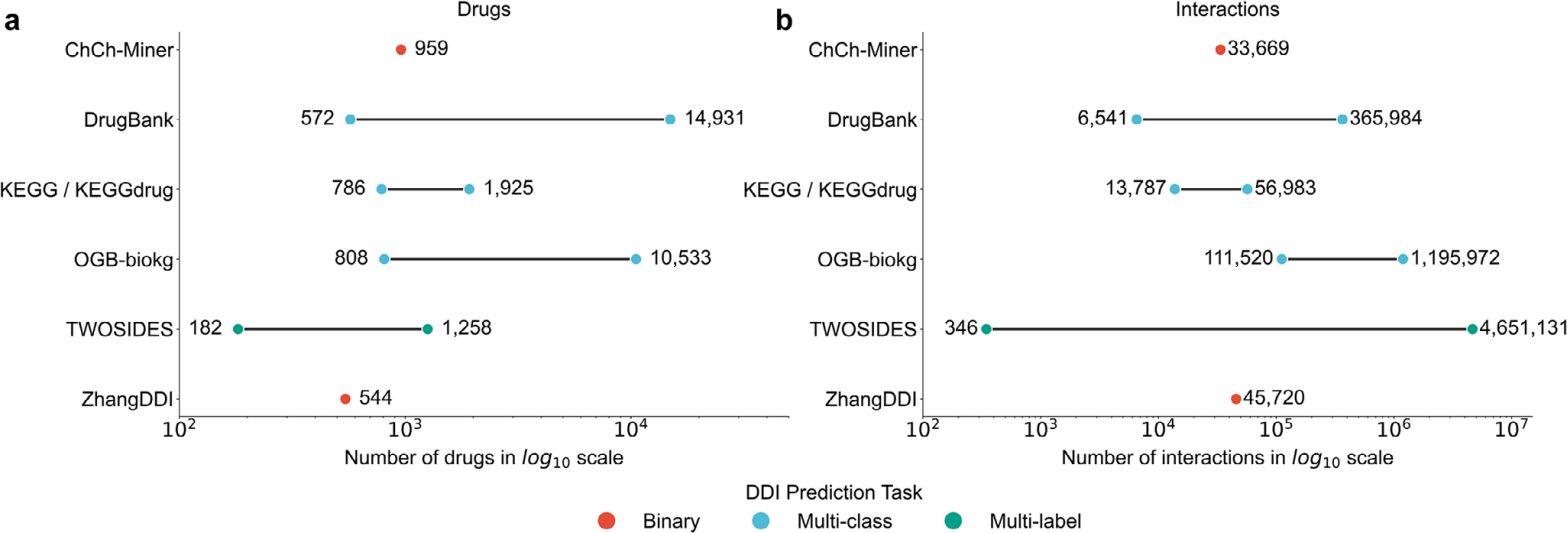
Statistical analysis of the widely used datasets for DDI prediction. (a) The variations of the number of drugs used in different models on the same dataset. (b) The variations of the number of interactions used in different models on the same dataset. In (a) to (b), the y axis shows the analysis for different datasets. Meanwhile, different colors indicate the specific DDI prediction tasks applicable to each dataset. The numbers are shown in logarithm scale for better visualization.

**FIGURE 7:**
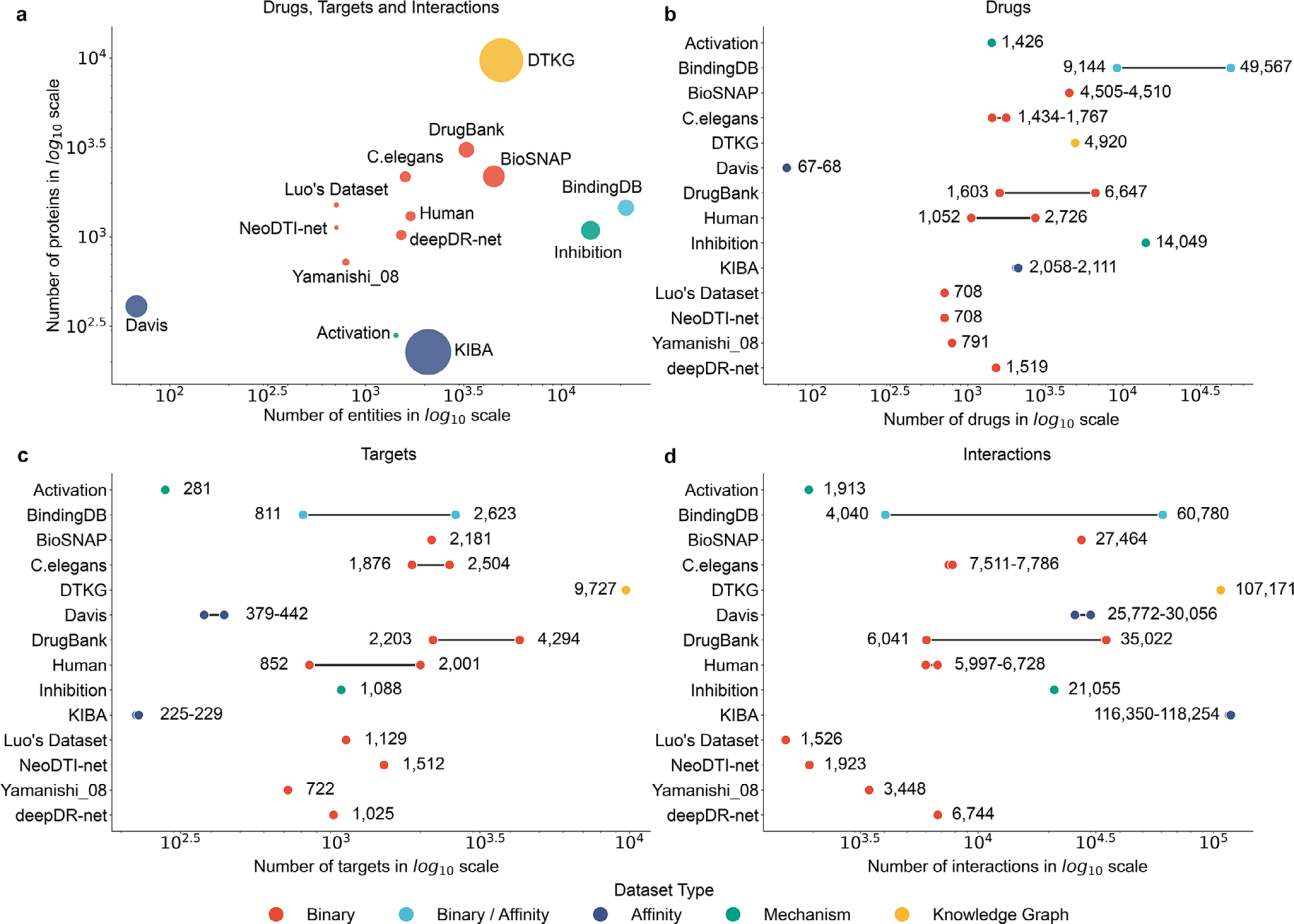
Statistical analysis of the widely used datasets for DTI prediction. (a) Visualization of the relationship among the number of proteins, the number of drugs and the number of interaction types for different DTI prediction datasets. For the same dataset, since different models select and use inconsistent numbers of entities and relationships, the number of proteins, drugs and interactions types of the same dataset is calculated as the geometric mean of the corresponding numbers across all models. (b) The variations of the number of drugs used in different models for the same dataset. (c) The variations of the number of targets used in different models for the same dataset. (d) The variations of the number of interaction types used in different models for the same dataset. In (a) to (c), the y axis shows the analysis for different datasets. In (a) to (d), different colors indicate the specific DTI prediction task applicable to each dataset. The numbers are shown in logarithm scale for better visualization.

Beyond the issue of treating unobserved interactions as false negatives, both domains suffer from label noise and inconsistency in experimental conditions. For instance, aggregating binding affinity data, ${\text{IC}}_{50}$ and $K_{i}$, from heterogeneous assays introduces high variance, resulting in a theoretical upper bound on achievable model accuracy that cannot be overcome by algorithmic improvements alone [120, 121, 122, 123]. Furthermore, distinct models usually employ diverse data curation strategies to produce different datasets, yielding varying dataset sizes. As shown in Figure 6, the number of entities in the same dataset utilized by various models for DDI prediction are dramatically different. This phenomenon is more noticeable on DrugBank dataset. The number of drug interactions range from 6,541 to 365,984, a difference of more than two orders of magnitude. Such inconsistency is primarily driven by arbitrary filtering criteria and various preprocessing strategies adopted by different studies. Therefore, directly comparing the reported performance of these models would introduce natural bias, obscuring their actual predictive capabilities. Furthermore, the DrugComb dataset serves as the gold standard benchmark for context‐dependent synergy tasks. However, performance evaluations in several studies are conducted on specific subsets, such as NCI‐ALMANAC dataset [124] and O'Neil dataset [125], leading to implicit data fragmentation. In Figure 7, we display the varying sizes of the same datasets adopted in different studies for DTI prediction, including Activation [78, 126], BindingDB [113], BioSNAP [109, 110], C.elegans [127], DTKG [89], Davis [128], DrugBank [105, 106], Human [127], Inhibition [78, 126], KIBA [129], Luo's Dataset [130], NeoDTI‐net [131], Yamanishi_08 [132, 133], and deepDR‐net [134]. These datasets can be applied for different types of tasks, such as binary prediction and affinity prediction. The differences result from divergent decisions, e.g., negative sampling ratios, activity thresholds, and data filtering criteria, leading to biased benchmarking across studies. Consequently, direct performance comparisons become flawed, as it is unclear whether performance improvements arise from model design innovations or from less challenging tasks by data curation. The lack of standardized preprocessing protocols creates unfair comparisons between models and algorithms. Therefore, a strict and standard data curation standard should be set, offering a full and clear pipeline for data preprocessing and filter for unified comparison. In addition, the “data silos” phenomenon in the pharmaceutical industry prevents access to diverse, cross‐institutional datasets [135]. Developing privacy preservation technologies and designing new learning schemes, such as federated learning [136] could alleviate this issue and facilitate performance improvements on generalizability and accuracy.

### 5.4 Multimodal Complexity

Integrating diverse data modalities, from 1D sequences and 2D graphs to precise 3D structures and multi‐dimensional omics profiles, offers great potential for improving prediction accuracy, while it also introduces practical challenges. The primary difficulty arise from the inherent heterogeneity of these different sources and formats of model inputs. Since each data type possess a distinct feature space, requiring customized encoders, the difficulty and complexity of the model design are inevitably increasing [137]. Furthermore, to effectively perform model fusion, three kinds of strategies are proposed, i.e., early fusion, intermediate fusion, and late fusion. Table 3 provides a systematic comparison among these kinds of strategies. It details their mechanisms and contrasts their advantages and limitations when applied to DDI and DTI prediction tasks. The results indicate that intermediate fusion has been selected as the dominant paradigm. While early and late strategies provide structural simplicity, they would struggle to process the heterogeneous data and complex modal interactions. In contrast, intermediate fusion offers an optimal balance for modeling complex drug–drug and drug–target interactions.

**TABLE 3:** Comparison of three kinds of multimodal data fusion strategies for DDI and DTI predictions, including early fusion, intermediate fusion, and late fusion.

Fusion Strategy	Mechanism Summary	Representative Method	Advantages	Limitations
Early Fusion	This strategy concatenates raw or shallow extracted features from different modalities into a single joint vector before feeding it into the main predictive network.	CTF‐DDI [49] (2024)	(1) Straightforward and clear strategy to be implemented. (2) All original information from the raw or shallow features to be retained. (3) Low computational cost for this operation.	(1) The curse of dimensionality due to the concatenation of multiple features. (2) Challenges in aligning features from heterogeneous data spaces. (3) Domination of the higher‐dimensional modalities during the fusion process. (4) Insufficient capture of complex cross‐modal interactions.
Intermediate Fusion	This strategy employs separate encoders to extract informative representations in their latent spaces for different modalities, followed by feature fusion layers for cross‐modal interaction.	RareDDIE [55] (2025) PHGL‐DDI [57] (2025) MeTDDI [48] (2024) EviDTI [65] (2025) MSI‐DTI [89] (2024)	(1) Flexibility in the integration of advanced model architectures and learning paradigms. (2) Effectiveness in capturing complex interactions across different modalities. (3) Utilization of modality‐specific optimal encoders to feed high‐quality features into the prediction module. (4) Noise suppression via the alignment of modalities in a shared latent space.	(1) High computational complexity arising from the specific model design. (2) Domination of gradient updates and training converge by simpler modalities. (3) Overfitting risk and shortcut learning tendency due to large parameter spaces.
Late Fusion	This strategy trains parallel, independent models for diverse modality combinations, subsequently aggregating their output logits through ensemble mechanisms.	SITAR [138] (2011) Zhang et al. [139] (2017)	(1) High robustness to missing modalities. (2) Flexibility in sub‐model design, training and optimization for respective modalities. (3) Clear revelation on the respective contributions of modalities to the final prediction.	(1) Failure in capturing cross‐modal interactions among sub‐models. (2) High Computational cost from multiple sub‐model deployment. (3) Potential misalignment between the ensemble mechanisms and decision boundaries, limiting the performance of upper bound.

### 5.5 Limited Interpretability

Most models display their interpretability through attention‐based visualization [39, 47, 48, 65, 80]. As static mappings of chemical substructures overlook the dynamic complexity of molecular binding and interactions, these visualizations frequently reflect learned statistical correlations rather than the intrinsic physical mechanisms, creating a disconnect between algorithmic explainability and physiological reality.

## 6 Prospection

While the independent evolution of DDI and DTI methods has yielded a rich variety of solutions, the continued segregation of these fields represents a missed opportunity for synergy. Through our comprehensive analysis, both fields share the common patterns, model development trends and challenges. Therefore, we believe that DDI prediction should be re‐framed as a systemic consequence of specific DTI profiles rather than an isolated task. To bridge this gap, novel models should be designed to learn drug–target affinity prediction and drug–drug interaction prediction tasks simultaneously. Rather than training separate encoders, a unified multi‐task model would share drug and protein encoders across all objectives. By training on multiple tasks in parallel, the model could fully exploit rich protein‐ligand binding information to enhance the prediction of system‐level drug interactions. To achieve it, developing a universal and physics‐informed molecular representation encoder is the first step. Rather than solely relying on 2D molecular structural representations, the generalized encoder should further integrate 3D conformational structures and 1D physicochemical properties into a unified latent space, ensuring the model to capture complex molecular principles for different downstream tasks. Second, build upon this unified encoder, we should design a decoding mechanism following real‐world biochemical mechanisms instead of purely employing prediction heads. Since many drug–drug interactions are usually downstream consequences of underlying drug–target binding mechanisms, the model architecture should explicitly reflect this causality. For instance, utilizing the intermediate latent representations from DTI binding affinity prediction as the prior information for DDI classification module would allow the network to ground its DDI predictions in specific target‐level events, thereby improving both accuracy and interpretability. Third, it is also essential to implement an adaptive joint optimization strategy when optimizing models under different tasks. In practice, directly summing mean squared error for DTI prediction and binary cross entropy for DDI prediction often induces gradient conflicts and negative transfer [140]. Integrating gradient projection techniques is indispensable to dynamically balance task‐specific gradients and ensure mutually beneficial parameter updates [141, 142]. Finally, with the well‐designed architecture, the richness of data becomes the focal point. Considering that various prediction tasks posses different data requirements, it is necessary to map one biological entity to relevant entities as much as possible. Here, a single drug can simultaneously anchor continuous binding‐affinity values to protein targets and discrete interaction types to other drugs. Then, we can explicitly trace how microscopic binding events cause macroscopic adverse clinical reactions, and observe how macroscopic clinical phenotypes perform feedback regulation on microscopic target affinities.

As data quality is as critical as model design, employing natural language processing could mine “dark data” from sources beyond current used datasets, including negative data and failed experiments from patents, thesis, and supplementary materials. Recovering this “dark data” would reduce model bias and provide a more realistic view of the chemical landscape, addressing the cold‐start problem by grounding predictions in a comprehensive pharmacological context. Building upon these, the recent developments of LLMs, such as BioGPT [143] and Med‐PaLM [144], offer great potential for the data‐mining paradigm. Different from traditional NLP methods that rely on rigid pattern matching, LLMs perform deep contextual reasoning, enabling them to parse massive volumes of unstructured biomedical literature and clinical reports [145]. Crucially, LLMs can extract implicit interaction relationships and latent pharmacological synergies that are not easily mined from unstructured databases. Specifically, consider a scenario where one study identifies a drug as an inhibitor for a signaling pathway, while another links the activation of the signaling pathway to the expression of a protein. Through cross‐document reasoning, LLMs can automatically synthesize such disparate information across documents to infer latent, indirect modulatory interactions. Furthermore, the ability of continuous learning [146] of LLMs enable them to uncover the underlying biological and chemical principles embedded within clinical reports. Consequently, LLMs evolve from passive recipients to active designers, proposing new drug pairs derived from the learned knowledge and offering highly promising candidates for subsequent biological validations.

In addition, the rapid development of generative models represents a promising frontier in AI‐driven drug discovery field. We envision generative frameworks capable of rational design, creating novel molecular structures that optimize a desired DTI profile while minimizing adverse DDI risks. However, since current generative models sometimes design novel molecules that are chemically valid but physically implausible in 3D space [147, 148], future research should incorporate physical constraints and geometric priors directly into the generation process. Notably, physics‐Informed Neural Networks (PINNs) [149, 150] and Machine Learning Force Fields (MLFFs) [151, 152] are promising solutions to these problems. Recent studies on PINNs and MLFFs have shown that integrating explicit physical priors into network designs and loss functions, such as energy landscapes, electrostatics, and long–range interactions, can effectively improve robustness and generalization of the model across chemical space. Xu et al. [153] proposed a PINN‐based machine learning potential framework that jointly optimizes different physical constraints, achieving reliable predictions for macroscopic diffusion properties with only limited training samples. ViSNet [99] leveraged a geometry‐enhanced equivariant GNN and physical informed vector‐scalar interaction strategy to describe the potential energy landscapes and atomic forces of different biology molecules. This architecture is further enhanced by integrating polarization effect modeling for non‐local interactions in ViSNet‐PIMA [100], enabling more accurate MLFF with essential long‐range physical interactions. SO3LR [154] combined SO3krates neural network with short‐range repulsion, long‐range electrostatic forces and dispersion, achieving transferable molecular simulations of various molecular systems. In parallel, 3D‐aware generative frameworks that encode pharmacophoric or interaction patterns demonstrate that integrating structural priors leads to ligands with more realistic geometries, stable binding poses, and better transfer to unseen targets [155, 156]. These advanced methods provide insightful and valuable instructions on how to design adequate constrains for generating active and physically reliable molecules.

Furthermore, future prediction models should shift from correlation to causation with unified biomedical knowledge, where DTI and DDI relations treated as interconnected components are constructed and updated. Current approaches often segregate these into different networks, thereby obscuring the causal link between target binding and drug interaction. By incorporating causal inference [157], models should evolve into interventional tools capable of addressing counterfactual questions, such as how modifying this functional group would alter the systemic DDI profile. Specifically, future models should integrate causal machine learning techniques, such as Structural Causal Models (SCMs) [158] or Pearl's do‐calculus [159], directly into the representation learning process. By defining the drug interaction meta‐path like $e_{i}^{\text{drug}}\to e_{m}^{\text{target}}\to \text{Pathway}\leftarrow e_{j}^{\text{drug}}$ within an SCM framework, researchers can simulate interventions actively. Using counterfactual reasoning strategies, a model could evaluate whether a predicted DDI would still occur if the binding affinity between drug $e_{i}^{\text{drug}}$ and target $e_{m}^{\text{target}}$ were artificially neutralized in the latent space. This paradigm shift from predictive modeling to rational, physically grounded design will shape the next generation of AI‐driven drug discovery.

## Author Contributions

T. W. led, conceived, designed, and supervised the study. X. S. conducted experiments and analysis. X. S. and T. W. wrote the original manuscript. All authors approved the final version of the manuscript.

## Conflicts of Interest

The authors declare no conflicts of interest.

## Data Availability

The data processing pipelines, dataset splits, and evaluation scripts used in self‐paired pseudo negative experiments for shortcut learning evaluation are available at https://github.com/WangGroup‐AI/AI4DrugInteraction.
